# The Physiological Cost of the Boat-Race

**Published:** 1896-04-04

**Authors:** 


### THE PHYSIOLOGICAL COST OF THE BOAT-RACE.

The Oxford and Cambridge boat-race of 1896 has been one of the most strenuous of recorded inter-university struggles. The race was won by two-fifths of a length. Only on four previous occasions in sixty-seven years has there been so narrow a margin of victory. The fight and its excitement are quite over, and it is worth while to consider, now that everybody's pulse doth once more "temperately keep time," whether the "game" was really "worth the candle."

With us the question is, of course, almost entirely one of physiology. We do not take the view that physiology includes the whole of man and his life: but it includes so very much that what is left over when every physiological consideration has been fully dealt with is of little account, at any rate in such a purely physical contest as that of a boat-race. The least excitable person could see last Saturday that no struggle could possibly have been keener or more tremendous whilst it lasted, even a struggle involving the issues of life and death. For the period of twenty minutes and one second every member of the two crews was at the highest physiological pitch of which his organism was capable. The strain fell principally upon the two main centres of life—the heart and the nervous system. Speaking as judicially as is possible, a physiologist cannot but express the conviction that every man in the two boats, except, perhaps, the two coxswains, must have been to some extent injured.

It is the habit of the Englishman to take his contests calmly. But if anyone supposes that the calm person subjects his nervous system to less strain than the more excitable, he is much mistaken. The mere effort to preserve outward calm is of itself a powerful exhauster of nervous energy. But in a contest like Saturday's it was impossible for any man in either of the boats to keep calm. The tremendous muscular efforts made set up such a swift and high-tensioned cardiac activity as could not but charge to overflowing every remotest capillary supplying the nervous system and caused a production of nerve energy which was the very highest of which the nervous portion of the organisation is capable. The nerve strain in such cases is intense, and the waste of nerve tissue is almost incredible.

It is not, however, the nervous system to which the most serious and enduring harm is done on occasions of this kind: it is the heart.

The question then arises, Ought we at this period of our civilisation to be indulging in contests of this kind? We do not say that no contests, even physical contests, are to be encouraged. On the contrary, we believe that contests are an essential part of human experience at every period of life. Moreover, contests must be real and strenuous, as strenuous as they can be made. But there is one factor in a twenty minutes' boat-race which we consider absolutely fatal to its value as a classical game. The factor is this, that it keeps up the most tremendous strain of which the human frame is capable at its highest possible pitch, and uninterruptedly, for a period which is longer than any human organism can bear without harm. That is the fatal factor in the boat-race. Football, by Rugby rules, is as strenuous as a boat-race; but its strenuousness is not continuous. It is interrupted every few minutes; the "scrummages" at football never last longer than a limited number of seconds, and never engage more than a limited number of each team. In the boat-race every oarsman, from start to finish, is at the extremest possible point of physiological tension without interruption. The price, then, we pay for our boat-races is a price we pay for no other University or school contest known to the whole history of games. It is certain that no race is ever run without serious injury to some. In a definite number of cases the injury is life-long to individuals; and in every race every man can hardly fail to be more or less of a sufferer for a longer or shorter time. Such is the game, and such is the candle. Is "the game worth the candle"? We do not think it is.

